# Soft Tissue Ewing Sarcoma Cell Drug Resistance Revisited: A Systems Biology Approach

**DOI:** 10.3390/ijerph20136288

**Published:** 2023-07-03

**Authors:** Seyedehsadaf Asfa, Halil Ibrahim Toy, Reza Arshinchi Bonab, George P. Chrousos, Athanasia Pavlopoulou, Styliani A. Geronikolou

**Affiliations:** 1 Izmir Biomedicine and Genome Center (IBG), 35340 Izmir, Turkey; sadaf.asfa@ibg.edu.tr (S.A.); ibrahimhaliltoy@gmail.com (H.I.T.); reza.arshinchi@ibg.edu.tr (R.A.B.); 2 Izmir International Biomedicine and Genome Institute, Dokuz Eylül University, 35340 Izmir, Turkey; 3 Clinical, Translational and Experimental Surgery Research Centre, Biomedical Research Foundation Academy of Athens, Soranou Ephessiou 4, 11527 Athens, Greece; chrousge@med.uoa.gr; 4 University Research Institute of Maternal and Child Health and Precision Medicine and UNESCO Chair on Adolescent Health Care, National and Kapodistrian University of Athens, Aghia Sophia Children’s Hospital, Levadeias 8, 11527 Athens, Greece

**Keywords:** Ewing sarcoma, anticancer drugs, bioinformatics, gene expression, epigenetic regulation, protein–protein interactions

## Abstract

Ewing sarcoma is a rare type of cancer that develops in the bones and soft tissues. Drug therapy represents an extensively used modality for the treatment of sarcomas. However, cancer cells tend to develop resistance to antineoplastic agents, thereby posing a major barrier in treatment effectiveness. Thus, there is a need to uncover the molecular mechanisms underlying chemoresistance in sarcomas and, hence, to enhance the anticancer treatment outcome. In this study, a differential gene expression analysis was conducted on high-throughput transcriptomic data of chemoresistant versus chemoresponsive Ewing sarcoma cells. By applying functional enrichment analysis and protein–protein interactions on the differentially expressed genes and their corresponding products, we uncovered genes with a hub role in drug resistance. Granted that non-coding RNA epigenetic regulators play a pivotal role in chemotherapy by targeting genes associated with drug response, we investigated the non-coding RNA molecules that potentially regulate the expression of the detected chemoresistance genes. Of particular importance, some chemoresistance-relevant genes were associated with the autonomic nervous system, suggesting the involvement of the latter in the drug response. The findings of this study could be taken into consideration in the clinical setting for the accurate assessment of drug response in sarcoma patients and the application of tailored therapeutic strategies.

## 1. Introduction

Sarcomas represent one-fifth of pediatric cancers and 1% of adult solid malignant tumors. They rise most frequently from connective tissues and only in one-tenth of cases from osseus tissues. Based on their tissue of origin, there are two main classes of sarcomas. The pathogenesis of sarcomas is still unclear, as amid established risk factors reside genetic and epigenetic causes. Literature evidence includes smoking, age, gestational age and weight, parental and maternal health status, occupational exposure to drugs, chemicals, etc. Unfortunately, the survival prognosis of sarcomas is rather poor [1].

At least seventy types of sarcomas have been described so far [2]. Ewing sarcoma is a rare type that develops in bones (skull, spine, pelvis, chest, legs, arms, feet) and soft tissues (head and neck, legs, retroperitoneum) equally frequently and within 13–20 years [3]. More specifically, the Ewing sarcoma family of tumors includes neoplasms localized in the chest wall; this type of peripheral primitive neuroectodermal cancer has been termed an Askin tumor. Other localized sarcomas include an extraosseous Ewing sarcoma (rising in tissues other than bone). Medical record of hernias strongly contributes to Ewing sarcoma [4,5,6].

The main symptomatology of sarcomas includes swelling and pain around the tumor; these symptoms may coincide with febrile episodes and unexplained bone breaks, while their treatment is designed after gene studies in tumor tissue. Treatment involves surgery, palliative care, chemotherapy options, stem cells, CAR-T cells, targeted therapy with a NEDD-8 inhibitor or a tyrosine kinase inhibitor, immunotherapy with immune checkpoint inhibitors, radiotherapy, etc. Chemotherapy is a widely used modality for the effective treatment of diverse types of cancers, including sarcomas. However, in many cases, cancer cells acquire resistance to chemotherapy, which poses a major problem as to the efficacy of the treatment [7,8], as described in detail by Nikolaou and colleagues (2018) [7,8].

Differential gene expression profiles are important indicators of cell resistance to anticancer treatments [9,10,11,12]. The differential expression of genes is often associated with epigenetic regulation by DNA methylation, histone modifications, or non-coding RNA species (ncRNAs), which affect gene expression without modifying the underlying DNA sequence [13,14,15]. There is compelling evidence that gene expression is regulated by ncRNA molecules at different levels [16,17,18]. MicroRNAs (miRNAs) are endogenous, small (~22 nucleotides in length), non-protein-coding RNAs that play a critical role in regulating the expression of target mRNAs at the post-transcriptional level by binding to complementary sequences on target mRNAs, the so-called miRNA response elements (MREs) [19]. MiRNAs reduce the stability of the target mRNAs and/or inhibit their translation, thereby downregulating the expression of the corresponding genes [20]. A single miRNA can potentially regulate the expression of multiple genes, and, conversely, a gene can be targeted by numerous miRNAs [21]. MiRNAs have been reported to play important regulatory roles in cancer drug resistance [22,23,24].

Lately, accumulating evidence points towards the key role of competing endogenous RNAs (ceRNAs) in miRNA-mediated gene regulation. CeRNAs are ncRNAs that share MREs with miRNAs, and, therefore, can sequester miRNAs (acting like ‘sponges’), preventing thus miRNAs from binding to their MREs and reducing their regulatory effect on target mRNAs [25,26,27].

Post-transcriptional miRNA-mediated crosstalk between long ncRNAs (>200 bp long) (lncRNAs) and mRNAs has also been reported in chemoresistance [28,29]. The lncRNAs can be capped, polyadenylated, and spliced, but they lack a functional open reading frame. These versatile molecules are implicated in a range of biological functions and cellular processes, including regulation of gene expression, cell–cell signaling, genomic instability, and RNA decay [17,18]. Notably, lncRNAs are largely involved in diverse cellular processes [17], including response to chemotherapies [30,31]. In this study, an integrated bioinformatics approach was applied to identify competing endogenous RNA networks—i.e., the miRNAs that regulate the expression of chemoresistance-related genes, and the lncRNAs that act as molecular sponges of those miRNAs.

The above evidence highlights the importance of identifying genetic and epigenetic biomarkers for chemoresistance in sarcomas. To this end, we have designed an analysis of ‘-omics’ data relevant to the response of A673 Ewing sarcoma cells to drug treatment, towards the identification of novel genetic biomarkers of drug response in sarcomas, as well as pivotal epigenetic regulators.

## 2. Materials and Methods

### 2.1. High-Throughput Gene Expression Data

The publicly available repository NCBI GEO (Gene Expression Omnibus) DataSets (https://www.ncbi.nlm.nih.gov/gds/; accessed on 23 October 2022) [32,33] was thoroughly searched for gene expression datasets related to sarcomas and drug treatment using the keywords: “drug” AND “sarcoma” AND (“human” or “homo sapiens”). The criteria for selecting datasets were: (i) gene expression data from treated and untreated human sarcoma tissues or cell lines, (ii) more than 5000 genes were included in the transcriptomic dataset. In this way, one eligible RNA-Seq dataset was obtained. The GEO series GSE118871 [34] (Table S1) includes the genome-wide gene expression of Ewing sarcoma A673 cells treated with SP-2509. This dataset contains cell lines both responsive and resistant to SP-2509. The drug-resistant cell lines (herein referred to as “resistant”) were established by exposing the corresponding parental A673 cells (untreated) to increasing concentrations of SP-2509 over a 7 month period (in increments of 100 nM) or 48 h (2 uM). Both the resistant cells and those responsive to drug treatment (hereinafter called “responsive”) were assessed by Pishas et al. [34] based on experimental cell viability assays; the resistant cells demonstrated a significantly increased viability as compared to parental A673 cells treated with SP-2509, whereas the responsive cells displayed a reduced viability following drug treatment (Table S1). The Illumina HiSeq 2000 (Homo sapiens) GPL11154 platform was employed.

### 2.2. RNA-Seq Data Processing and Identification of Differentially Expressed Genes

The FASTQ files that contain raw 2 × 50-bp paired-end RNA-Seq reads were downloaded from the respective Sequence Read Archive (SRA) using the SRA Tool Kit v.2.9.0 [35] with the *fastq-dump –gzip –skip-technical –readids –dumpbase –clip –split-3* command. Raw RNA-Seq reads were mapped to the human reference genome GRCh38 (Ensembl version 104) with the usage of the splice junction aligner HISAT2 v.2.1.0 [36] with the “hisat2 -p -dta -x {input.index} -U {input.fq} -S {out.sam}” parameters. The output SAM files were converted to the compressed binary BAM file with the usage of SAMtools v.1.14 [37] with the “samtools sort -@ 10 -o {output.bam} {input.sam}” commands. Transcriptome normalization, reconstruction, and quantification was conducted by employing the StringTie version 1.3.5 [38], with the “stringtie -e -B -p -G {input.gtf} -A {output.tab} -o {output.gtf} -l {input.label}{input.bam}” parameters. The assembled transcripts and their estimated abundances were included in the output GTF file.

To identify DEGs between resistant versus responsive, and responsive versus parental Ewing sarcoma cells, the EdgeR package version 3.32.0 [39] of the R statistical computation environment v.3.6.1 (https://www.r-project.org; accessed on 28 October 2022) was used. The negative binomial (NB) distribution was used to model the RNA-Seq read counts per gene per sample in EdgeR. Then, the estimating dispersion was calculated with the *estimateDisp* function. Differential expression analysis between the two RNA-Seq groups was performed using the *exactTest* function of the EdgeR package v3.32.0. For determining statistically significant DEGs, the cutoff for the absolute log_2_-fold change (FC) was set at two (|log_2_FC≥2|), and the Benjamini and Hochberg (BH)-adjusted *p*-value [40] was ≤0.05.

The official HUGO Gene Nomenclature Committee (HGNC) [41] gene symbols and gene names were used.

### 2.3. Gene Set Enrichment Analysis

Gene set enrichment analysis (GSEA), a method to identify biological terms that are enriched in a large gene set, was conducted to functionally annotate the drug resistance-associated genes detected in this study. To this end, the ‘resistant’ DEGs were provided as input to WebGestalt (WEB-based GEne SeT AnaLysis Toolkit) [42,43] to identify statistically significant over-represented Gene Ontology (GO) terms; the non-redundant Biological Process subontology of GO was selected, the threshold for the BH-corrected *p*-value [40] was set at 10^−3^; the hypergeometric distribution was applied.

### 2.4. Functional Association Network

The associations among the ‘resistant’ genes/proteins under study were investigated and visualized with the usage of the STRING (Search Tool for Retrieval of Interacting Genes/Proteins) v11.5 [44], a database of both experimentally derived or predicted, direct or indirect, association data among genes/proteins extracted from diverse resources. A relatively high confidence score (≥0.6) for displaying interactions was chosen. The associations were further investigated, analyzed, and visualized through the open-source platform Cytoscape (v.3.8.2) (https://cytoscape.org/; accessed on 11 December 2022) [45].

### 2.5. Epigenetic Regulators of Chemoresistant Genes

The ncRNAs—i.e., miRNAs and lncRNAs—likely regulating the ‘resistant’ DEGs under study were investigated by employing state-of-the-art software tools.

Both the experimentally supported and predicted miRNAs targeting the DEGs were collected with the usage of: (i) microT_CDS (http://diana.imis.athena-innovation.gr/DianaTools/index.php?r=microT_CDS/index; accessed on 7 January 2022) has implemented the target prediction algorithm DIANA-microT-CDS [46]; (ii) TargetScan (http://www.targetscan.org/vert_72/; accessed on 7 January 2022) searches for the presence of conserved 8mer, 7mer, and 6mer sites that match the seed region of each miRNA [47]; (iii) miRDB (http://mirdb.org/; accessed on 8 January 2022) predicts functional miRNA gene targets by machine learning modeling of target gene expression data [48,49]; (iv) PicTar (https://pictar.mdc-berlin.de/; accessed on 8 January 2022) includes published, experimentally validated miRNA targets [50]. The miRNA/mRNA associations from the four databases were integrated and the duplicates were removed.

The DIANA-LncBase v.3 (https://diana.e-ce.uth.gr/lncbasev3; accessed on 24 January 2022) [51], a comprehensive database of experimentally supported miRNA targets on non-coding transcripts, was employed to identify those lncRNAs that potentially interact with the miRNAs detected in the previous step. To increase the accuracy of our analysis, only the miRNA–lncRNA interactions with high confidence detected in cancer/malignant cell types were considered.

An in-house Python script was used to retrieve miRNA–mRNA and miRNA–lncRNA interactions from the respective databases.

## 3. Results

**Figure 1:**
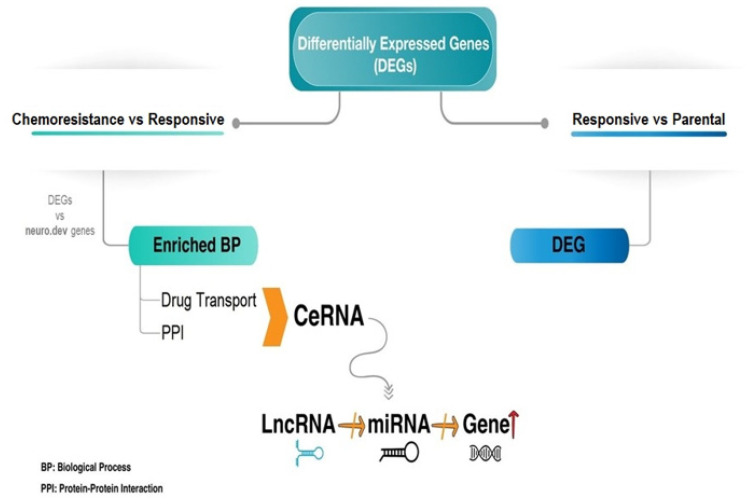
Graphical illustration of the overall methodology of this study. Genes differentially expressed between the chemoresistant and chemoresponsive, as well as the chemoresponsive versus parental Ewing sarcoma cells were detected. Functional annotation analysis of the genes upregulated in the chemoresistant cells and their protein products was performed. Subsequently, a ceRNA was generated from the drug resistance genes, their corresponding regulating miRNAs, and the lncRNAs that act as sponges of these miRNAs.

The overall procedure followed in this study is outlined in Figure 1.

### 3.1. Gene Expression Profiles of Ewing Sarcoma Drug Resistance

The differentially expressed genes (DEGs) detected between the chemoresistant and chemoresponsive, as well as the chemoresponsive versus the untreated parental Ewing sarcoma cells [34], were 1601 and 1196, respectively (Table S1). Among the genes found upregulated in the drug resistant cells were those encoding “drug resistance-associated membrane proteins” or “DRAMPs”, which affected the transport of drugs across membranes. The *ABCA2/8/9* genes belong to the broad ATP-binding cassette (ABC) transporter superfamily, the members of which pump drug molecules out of the cell, thereby decreasing the net accumulation of drugs within cancer cells. *ABCA2* and *ABCA9* were down-regulated in the responsive cells. The expression of a great number of genes coding for another class of DRAMPs, the solute carrier (SLC) transporters, which interfere with the translocation of drug molecules across membranes relying on physicochemical processes [52,53], was also dysregulated (Table S1). A key player in angiogenic signaling in cancer, the vascular epithelial growth factor, VEGFB, was overexpressed in resistant cells (Table S1). Treatment with specific VEGF inhibitors results in transient tumor vascular normalization and increased cancer cell response to chemotherapeutic drugs [54]. An increased expression of *ALDH1A3* and *ALDH1B1*—encoding aldehyde dehydrogenases (ALDH), detoxifying enzymes that catalyze the oxidation of intracellular aldehydes—was detected in the chemoresistant cell lines; ALDHs have been proposed as biomarkers of chemoresistance [55]. *ALDH1B1* was also under-expressed in the chemoresponsive cells.

Of note, non-common DEGs (with the same direction, either up- or down-regulated) were detected when the resistance versus responsiveness and responsiveness versus parental (untreated) cells were compared (Table S1). These findings indicate that our results were biologically meaningful, as different molecular factors and mechanisms were implicated in these two phenomena.

### 3.2. Functional Annotation Analysis

**Figure 2:**
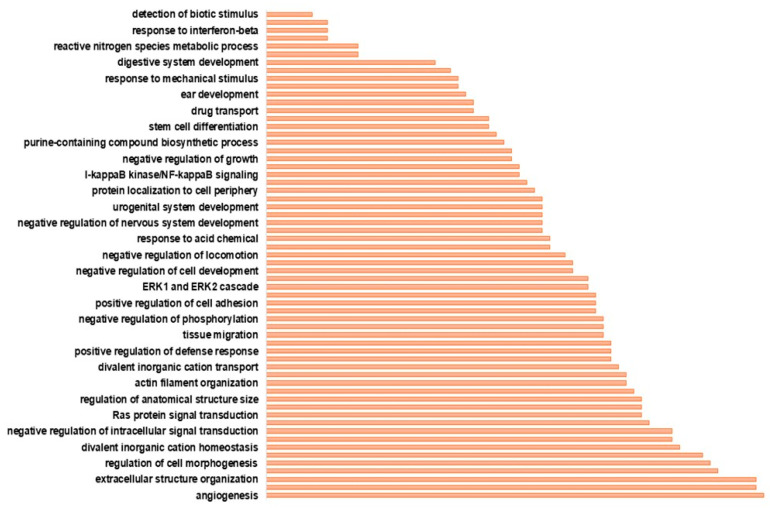
Bar plot depicting the over-represented biological processes (GO terms) in the chemoresistant genes. The *x*-axis corresponds to the number of genes associated with the GO terms; the length of the bars is proportional to the number of genes participating in the given biological process.

**Figure 3:**
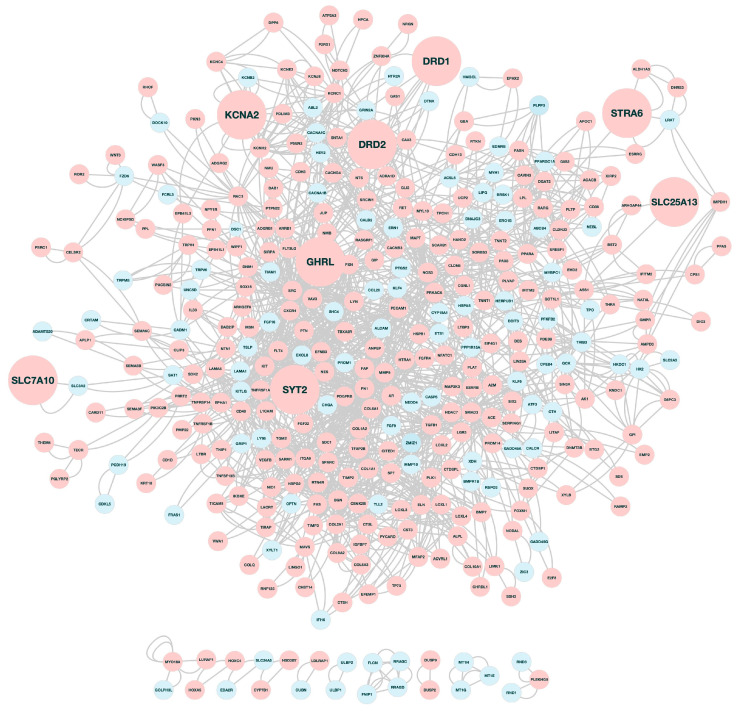
The STRING output interaction network of the resistance genes/proteins. The nodes represent genes/gene products, and the connecting lines denote functional associations. Pink and cyan fill colors indicate upregulation and downregulation of the corresponding genes, respectively.

A total of 641 ‘chemoresistant’ genes products were found to be implicated in known biological processes (Figure 2 and Table S2). The protein products of 369 (out of 641) genes formed a highly interconnected network (Figure 3 and Table S1), suggesting a functional association among these genes leading to their drug resistance effect. One of the over-represented biological processes was ‘drug transport’, which included 27 genes, 22 of which were up-regulated in the drug resistant cells (Figure 2 and Table S1). Given that an increased drug transport activity plays a critical role in drug resistance [7,56] and the bioentities that participate in networks are of a higher biological significance [57], we selected these eight over-expressed genes (*DRD1*, *DRD2*, *GHRL*, *KCNA2*, *SLC7A10*, *SLC25A13*, *STRA6*, *SYT2*), the protein products of which participate in the constructed network (Figure 3). Of note, the genes *DRD2*, *KCNA2*, *SLC7A10*, *STRA6*, and *SYT2* were under-expressed in the chemoresponsive versus untreated parental cells (Table S1).

### 3.3. Epigenetic Regulatory Network of Drug Resistance-Relevant Genes

To gain a glimpse into the post-transcriptional regulatory mechanism(s) of the eight SP-2509-resistant genes, a ceRNA network was constructed, consisting of the miRNAs that regulate the expression of the drug resistance genes, and the lncRNAs, capable of acting as molecular sponges of these miRNAs. For instance, several studies suggest that the downregulation of cancer-relevant miRNAs through sponging alleviates the suppression of downstream mRNAs, affecting different aspects of carcinogenesis [58,59,60].

**Figure 4:**
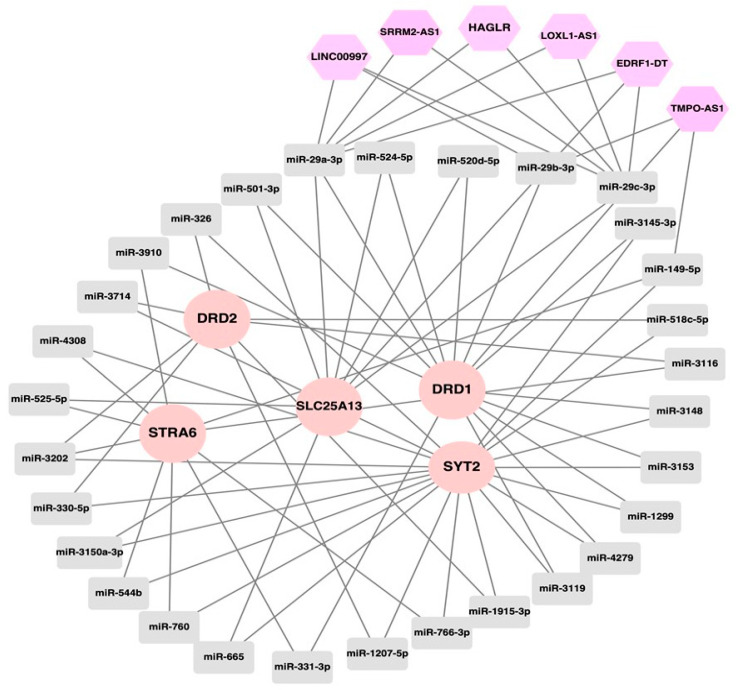
Competing endogenous RNA network in Ewing sarcoma resistance. The lncRNAs are represented by polygons, the miRNAs sponged by lncRNAs are denoted by rectangles, and the miRNA target genes are indicated by circles.

The miRNAs that potentially target the eight genes in Figure 3 were predicted using different methods. The computational methods for miRNA/mRNA target prediction usually depend on sequence-based features, thermodynamic stability, evolutionary information, or probabilistic models, etc. [61,62,63]. In our study, we used four tools based on different algorithms, so as to extract the maximum possible information. To enhance the prediction accuracy, only those miRNA–target gene interactions predicted by more than three tools were included in the study. Moreover, to obtain more robust results, miRNAs targeting more than two genes were selected for analysis. Collectively, 30 miRNAs were found to target more than 2 genes, and, conversely, 5 genes were targeted by more than 2 miRNAs (*DRD1/2*, *SLC25A13*, *STRA6*, *SYT2*), suggesting possible co-regulation at the post-transcriptional level. Of these miRNAs, based on our stringent selection criteria, 8 (hsa-miR-149-5p, hsa-miR-29a/b/c-3p, hsa-miR-330-5p, hsa-miR-501-3p, hsa-miR-760 and hsa-miR-766-3p) interacted with the lncRNAs in DIANA-LncBase (Table S2). We retained only the lncRNAs that were upregulated in the drug resistant cells—that is, co-upregulated with the target genes (Tables S1 and S2). Subsequently, a competing endogenous RNA network of the selected six lncRNAs (*EDRF1-DT*, *HAGLR, LINC00997*, *LOXL1-AS1*, *SRRM2-AS1*, *TMPO-AS1*), their sponged miRNAs, and the genes targeted by the miRNAs was constructed; however, the mirnas that interact with the DRD2 were not sponged by any lncRNAs (Figure 4).

The three members of the mir-29 family, miR-29a/b/c, were the ones mostly targeted by the lncRNAs (Figure 4). In particular, miR-29s have been reported to act primarily as tumor suppressors in numerous cancers through the upregulation of tumor suppressor genes or/and downregulation of oncogenes; they can, thus, elicit apoptosis and inhibit invasion and proliferation of cancer cells [64,65]. In a similar manner, they suppressed the activity of the SP-2509 resistance genes, resulting in the Ewing sarcoma cells responding to drugs. Conversely, the lncRNAs identified in this study could act as drivers of chemoresistance in sarcomas through their sponging role regarding miRNAs (Figure 4). In agreement with this, accumulating evidence suggests that *LOXL1-AS1* [66,67,68,69], *HAGLR* [70,71], *LINC00997* [72], and *TMPO-AS1* [73,74,75] contribute to carcinogenesis by acting as molecular sponges of miRNAs.

## 4. Discussion

In this work, we investigated the molecular determinants of the Ewing sarcoma cell’s line A673 response to diverse anti-neoplastic agents through an in silico approach. To this end, a relevant, publicly available gene expression dataset was used, in which Pishas and colleagues [34] treated Ewing sarcoma cells with increasing concentrations of SP-2509 to investigate the mechanisms underlying the resistance to SP-2509, a small molecular reversible inhibitor of LSD1 [76], in sarcoma. The lysine-specific demethylase 1 (LSD1), also known as KDM1A, demethylates histone 3 lysine 4 (H3K4), thereby acting either as a transcriptional co-repressor or as a transcriptional co-activator by catalyzing the demethylation of histone 3 lysine 9 (H3K9) [77,78]. LSD1 has been demonstrated to contribute to carcinogenesis via chromatin modification, as it promotes or represses the transcription of oncogenes or tumor suppressor genes, respectively [79,80,81,82]. The majority (76%) of the genes found to be differentially expressed between the SP-2509 resistant and responsive Ewing sarcoma cells were up-regulated (Table S1), suggesting that the overexpressed genes contributed the most to drug resistance. Of those, eight genes were implicated in drug transport and their corresponding protein products were components of a highly connected network (Figure 2 and Figure 3). These genes/proteins included the signaling receptor and transporter of retinol STRA6, the synaptotagmin 2, the potassium voltage-gated channel KCNA2, as well as the receptor ligands ghrelin and obestatin prepropeptide GHRL. The dopamine receptors DRD1 and DRD2, a class of G protein-coupled receptors, activated adenylyl cyclase to convert ATP into cyclic AMP (cAMP), a second messenger, and were implicated in the dopaminergic regulation of essential neurophysiological processes [83,84]. Two solute carrier (SLC) transporters, SLC25A13 and SLC7A10, were also upregulated. SLC transporters facilitated the influx of drug molecules into cells [85]. However, it has also been suggested that several SLC transporters can mediate bi-directional transport (i.e., both influx and efflux) [86].

### Autonomic Nervous System and Drug Resistance

The autonomic nervous system (ANS) regulates many bodily functions, participating in a major fashion in the maintenance of homeostasis and the body’s response to stress, and may, thus, influence carcinogenesis. On the other hand, members of the dopamine receptor (DR) family are upregulated in diverse cancers. A positive correlation was observed between a reduced cancer risk and neurodegenerative disorders, such as Parkinson’s disease or schizophrenia, where DR-targeting drugs are administered. Moreover, DR antagonists have displayed anticancer efficacy [87,88]. Amid the nodes involved in our network, SYT2 has been suggested to play a regulatory role in synaptic vesicle trafficking and in promoting metastasis in ovarian cancer [89]. In addition, polymorphisms in the gene encoding the neuropeptide GHRL have been associated with non-Hodgkin lymphoma [90]. Three more of the identified major hubs in our constructed interactome: (1) the ion transporter KCNA2, normally expressed in the central nervous system (CNS) [91]; (2) the *STRA6,* the expression of which was reported in the differentiating nervous system [92]; and (3), the SLC7A10, also called the astrocytic transporter (Asc-1), which has been proposed as a primary driver of the D-serine uptake in the CNS [93]. Taken together, the above findings suggested a link between drug resistance and nervous system function.

Of note, in a study by Gaynes et al., the CNS niche was shown to enhance chemoresistance in leukemia [94]. Additionally, denervation enhanced the effectiveness of chemotherapy in gastric cancer [95]. Finally, Logotheti and colleagues had suggested that genes implicated in neuronal function are activated in cancer cells [96]. As little is known on the potential effects of the ANS on cancer chemoresistance, we compared the up-regulated drug resistance-associated molecules identified herein (Table S1) with murine knockout genes associated with abnormalities in neuronal development, as reported by Logotheti et al. [96]. Of the 119 molecules found in common (Table 1), 40 were oncogenes, including 2 Ewing sarcoma specific genes—namely, the NK2 Homeobox 2 (NKX2) and FEV transcription factor (FEV) [97,98,99]. It has been reported that the NKX2 expression as a biomarker has a high sensitivity (100%), but a moderate specificity in cytologic specimens [97]. Yet, the Ewing sarcoma specificity increased when combined with CD99 [100]. The NKX2 is selectively expressed in the brain, thyroid gland, parathyroid glands, lungs, skin, and enteric ganglia, from prenatal development to adulthood, and has key functions at the interface of the brain, the endocrine, and the immune systems. It is highly expressed in mature limbic circuits related to context-dependent goal-directed patterns of behavior, social interaction and reproduction, as well as in fear responses [101].

The Nkx2-1 is indirectly involved in autonomic regulation as a major central target for angiotensinogen produced in the subfornical organ and directed to the hypothalamic paraventricular nucleus, where it is converted to angiotensin II [101]. Angiotensin II in the paraventricular nucleus contributes to the autonomic output and sympathetic nervous system excitation [102] and the so termed “enteric branch” (third autonomic branch beyond the sympathetic and parasympathetic ones) of the ANS, as well [103,104,105,106]. The peculiar behavior of the latter branch was initially described by Sternini (1997) [107]. Its association to obesity was first observed in animal models, followed by a meta-analysis in human bariatric surgery data [108], and then described in a preliminary molecular network involved in the “third type of obesity” [109]. Yet, concerning cancer, although this protein has been proposed as a cytostatic factor in certain cancer types, this has not been confirmed on a large scale [110].

*FEV* gene rearrangements have been observed in a fraction of Ewing sarcoma patients (3.5%) being linked to axial extraskeletal locations (mainly soft tissue), “older age at diagnosis and aggressive clinical behavior”, but retaining the classic Ewing sarcoma image [99].

Most of the rest are molecules associated with sensory (deafness, eye disorders) and motor impairments, cardiovascular diseases (i.e., valvulopathies, Brugada syndrome, hypertension), metabolic disorders, neurodegenerative disorders (Parkinson’s, Alzheimer, myoskeletal disabilities), encepalopathies and various syndromes, as shown in Table 1. In this table, some molecules are of unidentified function, meriting further research.

Furthermore, five genes involved in drug transport (*DRD1/2*, *SLC7A10*, *STRA6,* and *SYT2*) and the *SLC25A13* paralog, *SLC25A1*, (Figure 3), are related to neuronal development (Table 1, italicized), further supporting the emerging role of the ANS in drug resistance in chemotherapy.

## 5. Conclusions

By employing an interdisciplinary in silico methodology, we identified genes involved in drug resistance in sarcoma cells. A non-negligible fraction of these genes was also associated with the nervous system function and/or development. Of note, the study of the chemoresistance modification effect of the autonomic nervous system in cancer is an ongoing research field with interesting and unpredictable results. Furthermore, this study suggested that future investigations should be directed towards deciphering the crosstalk between the ceRNA and epigenetic regulation of interconnected protein-coding genes. LncRNAs may be considered potential targets for drug resistance based on their capability to attract and inhibit downstream miRNAs, influencing their ability to suppress chemoresistance—associated mRNAs. Both the protein–protein interaction and ceRNA networks comprise of an orchestrated multi-molecular mechanism, the dissection of which would enhance our understanding of the molecular determinants of cancer cell chemoresistance. Therefore, by disrupting the lncRNAs–miRNA interplay and by repressing the corresponding mRNAs, as well as by manipulating interactions between proteins encoded by chemoresistance-relevant genes, the drug resistance of cancer cells could be attenuated. This information can be utilized in the clinical setting for the design of combinatorial therapies, targeting both proteins and epigenetic regulatory factors, such as miRNAs and lncRNAs.

## Figures and Tables

**Table 1:** Common genes between the SP-2509 drug resistance associated genes and murine genes affecting nervous system development. Associated disease syndromes for each gene may be crosschecked in the GeneCards/disorders platform. Genes associated to neurological disorders are marked in blue, genes associated to tumorogenesis or malignancies are marked in red, whilst genes involved in neural and/or cancer entities are marked in purple.

Gene Symbol	Gene Name	Associated Disease/Syndrome
AADAT	alpha-aminoadipate aminotransferase	Detrusor Sphincter Dyssynergia and Huntington Disease
ACVRL1	activin A receptor like type 1	Telangiectasia, Hereditary Hemorrhagic, Type 2; Idiopathic Pulmonary Arterial Hypertension
AR	androgen receptor	Spinal And Bulbar Muscular Atrophy
ARAP3	Ankyrin Repeat And Plekstrin Homology Domains-Containing Protein 3	Neurofibromatosis 1
ARHGAP44	Rho GTPase Activating Protein 44	Hyperalphalipoproteinemia 2
ASS1	Argininosuccinate Synthase 1	Citrullinemia
ATP11A	ATPase Phospholipid Transporting 11A	COVID-19; leukodystrophy
BMP7	Bone Morphogenetic Protein 7	renal fibrosis, spondylolistheis,
CACNB3	Calcium Voltage-Gated Channel Auxiliary Subunit Beta 3	distal hereditary motor neuropathy 2
CC2D1A	Coiled-Coil And C2 Domain Containing 1A	Intellectual Developmental Disorder, Autosomal Recessive 3; Autosomal Recessive Non-Syndromic Intellectual Disability; cerebral palsy
CCKAR	Cholecystokinin A Receptor	panic disorder, functionless pituitary adenoma
CCNF	cyclin F	amyotrophic lateral sclerosis
CHAT	Choline O-Acetyltransferase	myasthenic syndrome, central sleep apnea, respiratory failure
CNTNAP1	ontactin Associated Protein 1	lethal congenital contracture syndrome, neuropathy
	Collagen Type II Alpha 1 Chain	epiphysial dysplasia
CRB2	Crumbs Cell Polarity Complex Component 2	Genetic Steroid-Resistant Nephrotic Syndrome; Ventriculomegaly With Cystic Kidney Disease
CST3	Cystatin C	kidney disease, Cerebral Amyloid Angiopathy, Cst3-Relate
CTSF	Cathepsin F	Neuronal Ceroid Lipofuscinosis; Spinocerebellar Ataxia
CXCR4	C-X-C Motif Chemokine Receptor 4	dementia; whim syndrome
DAB1	DAB Adaptor Protein 1	epilepsy; spinocerebral ataxia
DLX6	Distal-Less Homeobox 6	Isolated Split Hand-Split Foot Malformation
DMRTA2	DMRT Like Family A2	Uncertain
DNM1	Dynamin 1	Developmental And Epileptic Encephalopathy 31
DRD1	Dopamine Receptor D1	cerebral meningioma; Early-Onset Schizophrenia; Attention Deficit-Hyperactivity Disorder; heroin dependence
DRD2	Dopamine Receptor D2	Cocaine Dependence; drug deeondence; Antisocial Personality Disorder
DZIP1	DAZ Interacting Zinc Finger Protein 1	Orthostatic Intolerance; Mitral Valve Prolapse 3; spermatogenic failure
EEF1A2	Eukaryotic Translation Elongation Factor 1 Alpha 2	Developmental And Epileptic Encephalopathy
EEF2K	Eukaryotic Elongation Factor 2 Kinase	colorectal adenocarcinoma
EFNB3	ephrin beta3	Craniofrontonasal Syndrome
ENTPD1	Ectonucleoside Triphosphate Diphosphohydrolase 1	spastic paraplegia; Radiation Proctitis; beta-thalassemia
FAS	Fas Cell Surface Death Receptor	Autoimmune Lymphoproliferative Syndrome
FASN	Fatty Acid Synthase	NAFLD, prostate Ca
FBXL21P	F-Box And Leucine Rich Repeat Protein 21, Pseudogene	uncertain
FEV	FEV Transcription Factor	Ewing sarcoma; anxiety
FLT4	Fms Related Receptor Tyrosine Kinase 4	Congenital Heart Defects, Multiple Types, 7; Hereditary Lymphedema I; Hemangioma
FN1	fibronectin 1	soft tissue chondroma; Spondylometaphyseal Dysplasia; Spondyloepimetaphyseal Dysplasia
FRZB	Frizzled Related Protein	osteogenic sarcoma; retinal degeneration
GAL3ST1	Galactose-3-O-Sulfotransferase 1	Renal Cell Carcinoma, Nonpapillary
GAS1	Growth Arrest Specific 1	Septopreoptic Holoprosencephaly
GAS7	Growth Arrest Specific 7	Open-Angle Glaucoma; deafness; glaucoma
GBA	Glucosylceramidase Beta	Gaucher Disease, Type I,2,3,3c
GDF11	Growth Differentiation Factor 11	Vertebral Hypersegmentation And Orofacial Anomalies; aging
GLI2	GLI Family Zinc Finger 2	Holoprosencephaly 9; Combined Pituitary Hormone Deficiencies
HAND2	Heart And Neural Crest Derivatives Expressed 2	Cardiomyopathy, Dilated, 1a/h; Dilated Cardiomyopathy
HHEX	Hematopoietically Expressed Homeobox	Heart Defects, Congenital, And Other Congenital Anomalies; Diabetes Mellitus, Neonatal, With Congenital Hypothyroidism
HPCA	Hippocalcin	Dystonia 2, Torsion, Autosomal Recessive
HSPG2	Heparan Sulfate Proteoglycan 2	Dyssegmental Dysplasia, Silverman-Handmaker Type Tardive Dyskinesia
IL33	Interleukin 33	Chronic Asthma
INKA1	Inka Box Actin Regulator 1	Visual Cortex Disease
IQSEC2	IQ Motif And Sec7 Domain ArfGEF 2	Intellectual Developmental Disorder, X-Linked 1
ISL2	ISL LIM Homeobox 2	Amme complex
KIRREL3	Kirre Like Nephrin Family Adhesion Molecule 3	Autosomal Dominant Non-Syndromic Intellectual Disability; Familial Nephrotic Syndrome; Granulomatous Disease
KNDC1	Kinase Non-Catalytic C-Lobe Domain Containing 1	Brugada syndrome
L1CAM	L1 Cell Adhesion Molecule	Corpus Callosum, Partial Agenesis Of, X-Linked; hydrocephaly
LAMA5	Laminin Subunit Alpha 5	Vitreous Detachment; Presynaptic Congenital Myasthenic Syndromes; Lama5-Related Multisystemic Syndrome
LHX6	LIM Homeobox 6	Waardenburg Syndrome, Type 2c; tooth ageenesis
LIMK1	LIM Domain Kinase 1	Supravalvular Aortic Stenosis
LMNB1	Lamin B1	Hutchinson-Gilford Progeria Syndrome; Leukodystrophy, Demyelinating, Adult-Onset
LYNX1	Ly6/Neurotoxin 1	Malde Meleda; Ovarian Serous Cystadenocarcinoma; amblyopia
MAPT	Microtubule Associated Protein Tau	Frontotemporal Dementia; Supranuclear Palsy,; parkinsson disease; parkinsson-dementia syndrome
MARVELD1	MARVEL Domain Containing 1	Facial Nerve Disease; Facial Paralysis; Heart Fibrosarcoma; Cranial Nerve Disease
MAVS	Mitochondrial Antiviral Signaling Protein	hepatitis; Newcastle diseases; Oropouche Fever
MMP9	Matrix Metallopeptidase 9	Metaphyseal Anadysplasia; Central Nervous System Tuberculosis; Brain Glioblastoma Multiforme; internal hemoroids
NAT8L	N-Acetyltransferase 8 Like	N-Acetylaspartate Deficiency; Canavan Disease; microcephaly; Miliaria Rubra
NCKIPSD	NCK Interacting Protein With SH3 Domain	Wiskott-Aldrich Syndrome; thrombocytopenia; leucemia, spheroid syndrome
NDRG2	NDRG Family Member 2	Charcot-Marie-Tooth Disease, Glioblastoma, Meningioma
NES	nestin	Medulloepithelioma; Central Neurocytoma; Optic Nerve Glioma; Periventricular Leukomalacia
NFIX	Nuclear Factor I X	Malan Syndrome; Marshall-Smith Syndrome; megalocornea
NKX2-2	NK2 Homeobox 2	Maturity-Onset Diabetes Of The Young; Oligodendroglioma; Ewing Sarcoma
NODAL	Nodal Growth Differentiation Factor	Wolff–Parkinson–White Syndrome; heart disease
NOS3	Nitric Oxide Synthase 3	stroke; alzheimer; hypertention, preeclampsia
NOTCH3	Notch Receptor 3	Cerebral Arteriopathy, Autosomal Dominant, With Subcortical Infarcts And Leukoencephalopathy
NPY1R	Neuropeptide Y Receptor Y1	Body Mass Index Quantitative Trait Locus 11
NTN1	netrin 1	Mirror Movements 4/1, Superior Semicircular Canal Dehiscence, Colorectal Ca
OTX1	Orthodenticle Homeobox 1	Agnathia-Otocephaly Complex, epilepsy, Bardet-Biedl Syndrome 15; dyslexia
PDGFRB	Platelet Derived Growth Factor Receptor Beta	Kosaki Overgrowth Syndrome, premature aging syndrome,
PIP5K1C	Phosphatidylinositol-4-Phosphate 5-Kinase Type 1 Gamma	Neurogenic Bladder; alcohol use disorder; Cerebellar Ataxia, Cayman Type
PITX1	Paired Like Homeodomain 1	Clubfoot, Congenital, With Or Without Deficiency Of Long Bones And/Or Mirror-Image Polydactyly, Clubfoot
PMP22	Peripheral Myelin Protein 22	Charcot-Marie-Tooth Disease And Deafness, Neuropathy, Hereditary, With Liability To Pressure Palsies, Guillain-Barre Syndrome
PRPH	peripherin	Frontotemporal Dementia And/Or Amyotrophic Lateral Sclerosis 3; Amyotrophic Lateral Sclerosis 19
PRRT2	Proline Rich Transmembrane Protein 2	Episodic Kinesigenic Dyskinesia 1, Prrt2-Associated Paroxysmal Movement Disorders, Convulsions, Familial Infantile, With Paroxysmal Choreoathetosis
RASGRF1	Ras Protein Specific Guanine Nucleotide Releasing Factor 1	myopia, Degenerative Myopia, Transient Neonatal Diabetes Mellitus
RET	Ret Proto-Oncogene	Pheochromocytoma, Multiple Endocrine Neoplasia, Thyroid Carcinoma
RNF165	Ring Finger Protein 165	uncertaın
RTN4R	Reticulon 4 Receptor	spinal cord injury, schizophrenia, psychotic disorder, leucemia
S1PR2	Sphingosine-1-Phosphate Receptor 2	deafness, pulmonary edema
SARM1	Sterile Alpha And TIR Motif Containing 1	Wallerian Degeneration, Retinoschisis 1
SCTR	Secretin Receptor	Gastrinoma, Jejunal Somatostatinoma, pancreas disease, Primary Biliary Cholangiti
SEMA3F	semaphorin 3F	neuroma, megacolon, lung Ca, tongue carcinoma
SEMA6B	Semaphorin 6B	epilepsy
SH3TC2	SH3 Domain And Tetratricopeptide Repeats 2	Genetic Motor Neuron Disease, Charcot-Marie-Tooth
SLC18A3	Solute Carrier Family 18 Member 3	Myasthenic Syndrome, Fetal Akinesia Deformation Sequence 1
SLC19A3	Solute Carrier Family 19 Member 3	Thiamine Metabolism Dysfunction Syndrome 2, Infantile Spasms-Psychomotor Retardation-Progressive Brain Atrophy-Basal Ganglia Disease Syndrome
SLC25A1	Solute Carrier Family 25 Member 1	Myasthenic Syndrome,, Combined D-2- And L-2-Hydroxyglutaric Aciduria
SLC7A10	Solute Carrier Family 7 Member 10	Hypotonia-Cystinuria Syndrome
SMARCC2	SWI/SNF Related, Matrix Associated, Actin Dependent Regulator Of Chromatin Subfamily C Member 2	Neurilemmomatosis
SRCIN1	SRC Kinase Signaling Inhibitor 1	Kohlschutter-Tonz Syndrome
SRRM3	Serine/Arginine Repetitive Matrix 3	deafness, burst Ca
STRA6	Signaling Receptor And Transporter Of Retinol STRA6	Microphthalmia, Syndromic 9
SYT2	Synaptotagmin 2	Myasthenic Syndrome, Congenital, 7a, Presynaptic, And Distal Motor Neuropathy; Lambert-Eaton Myasthenic Syndrome
TCF7L1	Transcription Factor 7 Like 1	Brust Ca, colorectal Ca, heaptocellular Ca, Arrhythmogenic Right Ventricular Cardiomyopathy
TEAD2	TEA Domain Transcription Factor 2	Multiple Acyl-Coa Dehydrogenase Deficiency; spastic paraplegia
THRA	Thyroid Hormone Receptor Alpha	Hypothyroidism, Resistance To Thyroid Hormone Due To A Mutation In Thyroid Hormone Receptor Alpha, Hyperthyroxinemia, bone giant cell tumor
TLE5	TLE Family Member 5, Transcriptional Modulator	Arthrogryposis, Distal
TNFRSF1A	TNF Receptor Superfamily Member 1A	Charge syndrome, periodic fever
TNFRSF1B	TNF Receptor Superfamily Member 1B	Psoriatic Arthritis, Rheumatoid Arthritis, Juvenile Rheumatoid Arthritis
TP73	Tumor Protein P73	respiratory failure, Oligodendroglioma, neuroblastoma
TRIM71	Tripartite Motif Containing 71	all types hydrocephalus, Autosomal Recessive Limb-Girdle Muscular Dystrophy Type 2h
TRPV4	Transient Receptor Potential Cation Channel Subfamily V Member 4	metatropic dysplasia, Hereditary Motor And Sensory Neuropathy
TSKU	Tsukushi, Small Leucine Rich Proteoglycan	miliaria
TUBB4A	Tubulin Beta 4A Class IVa	cerebral palsy, torsion dystonia, nervous system disease
UCP2	Uncoupling Protein 2	Hyperinsulinism Due To Ucp2 Deficiency
VAV3	Vav Guanine Nucleotide Exchange Factor 3	ovarian Ca, meningioma, glaucoma
VAX1	Ventral Anterior Homeobox 1	microphalmia
VEGFB	Vascular Endothelial Growth Factor B	Macular Retinal Edema; Macular Degeneration, Age-Related, 1
VWA1	Von Willebrand Factor A Domain Containing 1	Neuropathy, Hereditary Motor, With Myopathic Features; Neuromuscular Disease
WDR62	WD Repeat Domain 62	Congenital Nervous System Abnormality
ZIC1	Zic Family Member 1	Craniosynostosis 6
ZMIZ1	Zinc Finger MIZ-Type Containing 1	Neurodevelopmental Disorder With Dysmorphic Facies And Distal Skeletal Anomalies; Syndromic Intellectual Disability; Brain Stem Infarction

## Data Availability

All data and analysis methodologies are contained in the manuscript. Any additional data requests can be addressed to the corresponding authors.

## Supplementary Materials

The following supporting information can be downloaded at: https://www.mdpi.com/article/10.3390/ijerph20136288/s1, Table S1: Gene expression data analyzed in this study. Samples from the GSE118871 transcriptomic dataset; differentially expressed genes (resistant versus responsive, responsive versus parental); Table S2: Functional analysis of the Ewing chemoresistant genes.
